# The *Kcnq1ot1* Long Non-Coding RNA Affects Chromatin Conformation and Expression of *Kcnq1*, but Does Not Regulate Its Imprinting in the Developing Heart

**DOI:** 10.1371/journal.pgen.1002956

**Published:** 2012-09-20

**Authors:** Lisa Korostowski, Natalie Sedlak, Nora Engel

**Affiliations:** Fels Institute for Cancer Research/Biochemistry, Temple University School of Medicine, Philadelphia, Pennsylvania, United States of America; University of Cambridge, United Kingdom

## Abstract

Although many of the questions raised by the discovery of imprinting have been answered, we have not yet accounted for tissue- or stage-specific imprinting. The *Kcnq1* imprinted domain exhibits complex tissue-specific expression patterns co-existing with a domain-wide *cis*-acting control element. Transcription of the paternally expressed antisense non-coding RNA *Kcnq1ot1* silences some neighboring genes in the embryo, while others are unaffected. *Kcnq1* is imprinted in early cardiac development but becomes biallelic after midgestation. To explore this phenomenon and the role of *Kcnq1ot1*, we used allele-specific assays and chromosome conformational studies in wild-type mice and mice with a premature termination mutation for *Kcnq1ot1*. We show that *Kcnq1* imprinting in early heart is established and maintained independently of *Kcnq1ot1* expression, thus excluding a role for *Kcnq1ot1* in repressing *Kcnq1*, even while silencing other genes in the domain. The exact timing of the mono- to biallelic transition is strain-dependent, with the CAST/EiJ allele becoming activated earlier and acquiring higher levels than the C57BL/6J allele. Unexpectedly, *Kcnq1ot1* itself also switches to biallelic expression specifically in the heart, suggesting that tissue-specific loss of imprinting may be common during embryogenesis. The maternal *Kcnq1ot1* transcript is shorter than the paternal ncRNA, and its activation depends on an alternative transcriptional start site that bypasses the maternally methylated promoter. Production of *Kcnq1ot1* on the maternal chromosome does not silence *Cdkn1c*. We find that in later developmental stages, however, *Kcnq1ot1* has a role in modulating *Kcnq1* levels, since its absence leads to overexpression of *Kcnq1*, an event accompanied by an aberrant three-dimensional structure of the chromatin. Thus, our studies reveal regulatory mechanisms within the *Kcnq1* imprinted domain that operate exclusively in the heart on *Kcnq1*, a gene crucial for heart development and function. We also uncover a novel mechanism by which an antisense non-coding RNA affects transcription through regulating chromatin flexibility and access to enhancers.

## Author Summary

Imprinted genes express only one copy and the active allele depends on the parental origin. These genes are usually organized in groups and are subject to regulation over long distances. Misregulation in these domains can lead to developmental diseases and cancer. The paternally expressed *Kcnq1ot1* non-coding RNA emerges from an intron of the *Kcnq1* gene in antisense direction and silences several neighboring genes. The *Kcnq1* gene is initially imprinted but becomes biallelic during development of the heart. To understand the mechanisms underlying this behavior, we performed expression and conformational studies of the *Kcnq1* domain during this transition and found that the early stage imprinting of *Kcnq1* is not dependent on transcription of *Kcnq1ot1*, thus revealing the existence of a distinct mechanism of silencing of the paternal *Kcnq1* allele. Surprisingly, *Kcnq1ot1* loses its imprinted expression specifically in the heart in the same time frame as *Kcnq1*. Absence of *Kcnq1ot1* does lead to overexpression of *Kcnq1* in later cardiac development, an event characterized by increased chromatin flexibility allowing the *Kcnq1* promoter to establish ectopic regulatory contacts. Thus, we have uncovered novel regulatory mechanisms that will help our understanding of tissue specificity in imprinted domains and how they can go awry in disease.

## Introduction

The genome is transcribed into a host of non-coding RNAs, some of which are designated as long intergenic (linc) RNAs. These have regulatory roles in gene activation and repression, and have been hypothesized to serve as guides for non-sequence-specific histone modifying enzymes [1]. One class of lincRNAs includes macroRNAs, which can range in size up to hundreds of kilobases. Several macroRNAs have been studied intensively in the fields of genomic imprinting and X-inactivation [2], [3], [4], [5]. The role of *Xist* in X-inactivation has been a long-standing paradigm for understanding how silencing of imprinted genes is achieved in domains that encode other macroRNAs. One prevalent model of imprinted gene silencing is that the non-coding (nc) RNA spreads along the chromatin, recruiting histone modifying repressive enzymes and rendering the region refractory to transcriptional activation.

The *Kcnq1ot1* imprinted ncRNA is transcribed from the paternal allele, emerging from intron 11 of the *Kcnq1* gene in antisense direction. In contrast to most long ncRNAs, *Kcnq1ot1* is highly expressed and detectable in every tissue examined [3], [6]. *Kcnq1ot1* transcription has a role in repressing several neighboring genes in *cis* [7], [8]. In both the embryo and the placenta, the polycomb group complex is reported to play a major role in silencing. In the placental lineage, repressive histone modifications are necessary to maintain the imprinting status of the genes surrounding *Kcnq1ot1* in the absence of differentially methylated regions [9]. In the embryo, only genes with stable DNA methylation are silenced by the ncRNA [10], [11], [12]. Although the role of *Kcnq1ot1* in the establishment of allelic repression is still unclear, evidence has emerged suggesting that maintenance of silencing results from the act of transcription through the locus rather than as a function of the RNA molecule *per se*, at least in cultured embryonic and trophoblastic cells [13].

The silencing capacity of *Kcnq1ot1* across the *Kcnq1* domain is not universal. At least one gene, *Cdkn1c*, is repressed by a mechanism independent of *Kcnq1ot1* in certain tissues [14]. On the other hand, some genes in the domain escape silencing, either ubiquitously or in a tissue-specific manner [15], [16], [17]. *Kcnq1*, one such exception, is expressed from the maternal allele during early embryogenesis and transitions to biallelic expression during fetal heart development. This transition coincides with conformational changes involving the interaction between the *Kcnq1* promoter and heart-specific enhancers [18]. Based on our previous studies, we hypothesized that paternal expression of *Kcnq1ot1* represses *Kcnq1* in *cis*, but this effect is countered when enhancer-driven activity of *Kcnq1* becomes established during heart development.

Defects in KCNQ1 are responsible for congenital long QT syndrome, a cardiac disorder that can result in serious cardiac arrhythmias [19]. There is a wide range of phenotypes, with some individuals remaining mostly asymptomatic and others presenting severe symptoms. Understanding the molecular mechanisms underlying the phenotypic variability of this disease will require fully elucidating the epigenetic profile of *Kcnq1* expression during cardiac development. We investigated function of *Kcnq1ot1* transcription in regulating *Kcnq1* in the heart in wild-type mice and in a mutant mouse model in which the *Kcnq1ot1* transcript is truncated. Our studies show that imprinted *Kcnq1* expression in early heart development is not dependent on the *Kcnq1ot1* transcript or on its transcriptional status. In fact, *Kcnq1ot1* itself suffers a loss of imprinting during cardiac development, although it remains monoallelic in other tissues. We also show that the ncRNA does have a role in later embryogenesis for proper regulation of *Kcnq1* levels in the embryonic heart by way of modulating the three-dimensional chromatin structure of the locus.

## Results

### The *Kcnq1ot1* ncRNA loses imprinted expression in the heart

**Figure 1:**
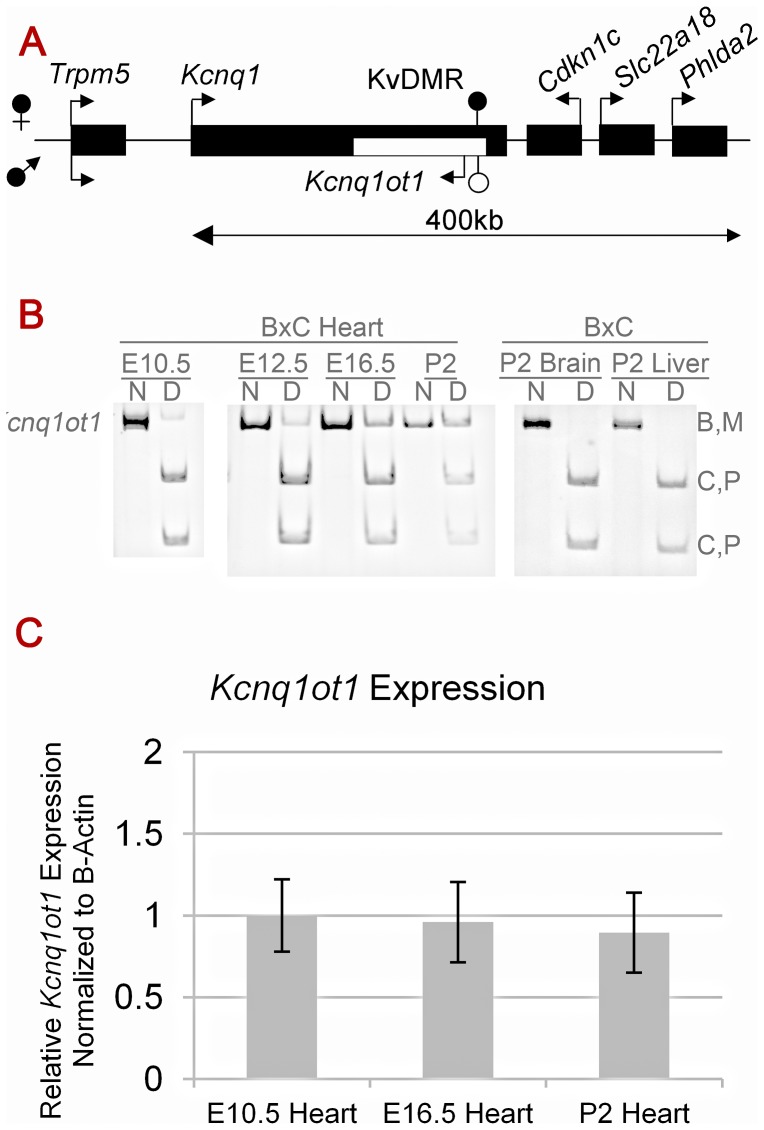
Cardiac expression profile of *Kcnq1ot1*. A) Imprinting pattern in the *Kcnq1* domain in early development in the embryo. Arrows above the line represent maternal transcription and those below represent paternal transcription. B) *Kcnq1ot1* imprinting as determined by RT-PCR followed by allele-specific restriction digest. The maternal *Kcnq1ot1* becomes activated as development progresses in the heart. However, in brain and liver, *Kcnq1ot1* remains monoallelic. M, maternal; P, paternal; N, non-digested; D, Digested; B, C57BL/6J; C, B6(CAST7). C) Quantitative analysis by qRT-PCR of *Kcnq1ot1* expression throughout development. RNA levels were normalized to *β-actin*.

The *Kcnq1* domain exhibits complex expression and imprinting patterns, eliciting questions of how long-range regulatory mechanisms coexist with locally limited ones (Figure 1A). The *Kcnq1* gene switches from mono- to biallelic expression during mid-gestation in a tissue-specific manner. In the embryonic heart the transition occurs at approximately E14.5, coinciding with enhancers contacting the *Kcnq1* promoter [18]. The timing for this is very consistent, with the switch occurring at the earliest at E13.5 and no later than E15.5. We proposed that tissue-specific enhancers compete with *Kcnq1ot1* and create a chromatin environment that is antagonistic to the ncRNA.

Activation of the paternal *Kcnq1* allele could interfere with spreading of the antisense *Kcnq1ot1* RNA, or it could impact its expression, thus limiting its role in silencing. To determine if the paternal *Kcnq1ot1* allele is still expressed after paternal *Kcnq1* activation, we carried out allele-specific RT-PCRs during normal heart development in progeny from reciprocal crosses between C57BL/6J and B6(CAST7) mice. B6(CAST7) are a consomic strain in which chromosome 7 is derived from *Mus musculus castaneus* [20]. Unexpectedly, we found that not only does the paternal *Kcnq1ot1* remain active, but the maternal allele is now transcribed as well. This loss of imprinted expression for *Kcnq1ot1* is independent of strain-specific effects, is specific to the heart and does not occur in other tissues (Figure 1B, Figure S1A).

Previous reports have shown that the paternal *Kcnq1ot1* ncRNA acts in *cis* by coating the chromosome [12], [11]. We used dual DNA and RNA fluorescent *in situ* hybridization (FISH) to determine if the maternal *Kcnq1ot1* transcript accumulates locally in the heart. RNA and DNA FISH was performed in primary cardiomyocytes that are biallelic for *Kcnq1ot1* (Figure S2). Results show that the maternal transcript co-localizes with the DNA strand it is being transcribed from. This suggests that the reactivated maternal *Kcnq1ot1* transcript does associate with the chromatin in *cis*.

As shown in Figure 1C, levels of *Kcnq1ot1* mRNA emerging from the maternal allele almost equal the paternal levels, suggesting that maternal expression is not merely due to “leaky” transcription. In fact, the transition to biallelic expression results in an increase in total *Kcnq1ot1* expression when compared to tissues in which *Kcnq1ot1* remains monoallelic (Figure S1B), Thus, the paternal activation of *Kcnq1* does not interfere with *Kcnq1ot1* transcription in *cis* [18] and, furthermore, initiation of *Kcnq1ot1* expression from the maternal allele does not silence the already active maternal *Kcnq1* allele.

Our data clearly indicate that cardiac development is accompanied by regulatory events in the *Kcnq1* domain that are very distinct from its expression and imprinting status in other tissues. To determine if the loss of imprinting in the heart affected genes in the region other than *Kcnq1* and *Kcnq1ot1*, we tested the allele-specific expression of *Cdk1nc* and *Slc22a18* using either RT-PCR followed by allele specific restriction digest or sequencing analysis (Figure S3). Interestingly, both *Cdkn1c* and *Slc22a18* maintained their maternal expression patterns throughout heart development. This confirms that transcription of the maternal allele of *Kcnq1ot1* that occurs in the heart does not have repressive capability. As anticipated, and as previously reported [14], *Cdkn1c* and *Slc22a1*8 maintain their imprinting expression in both P2 brain and liver samples (Figure S2A, S2B).

### The maternal *Kcnq1ot1* ncRNA emerges from an alternative promoter and is shorter than its paternal counterpart

The promoter of the *Kcnq1ot1* gene lies immediately upstream of two CG-rich regions that harbor a maternal-specific gametic methylation mark (KvDMR). To determine if activation of the maternal *Kcnq1ot1* allele is due to loss of methylation at the KvDMR, we performed methylation-specific PCR (data not shown) and bisulfite mutagenesis sequencing (Figure S4). Our results show that the maternal KvDMR methylation mark is not lost in neonatal heart.

**Figure 2:**
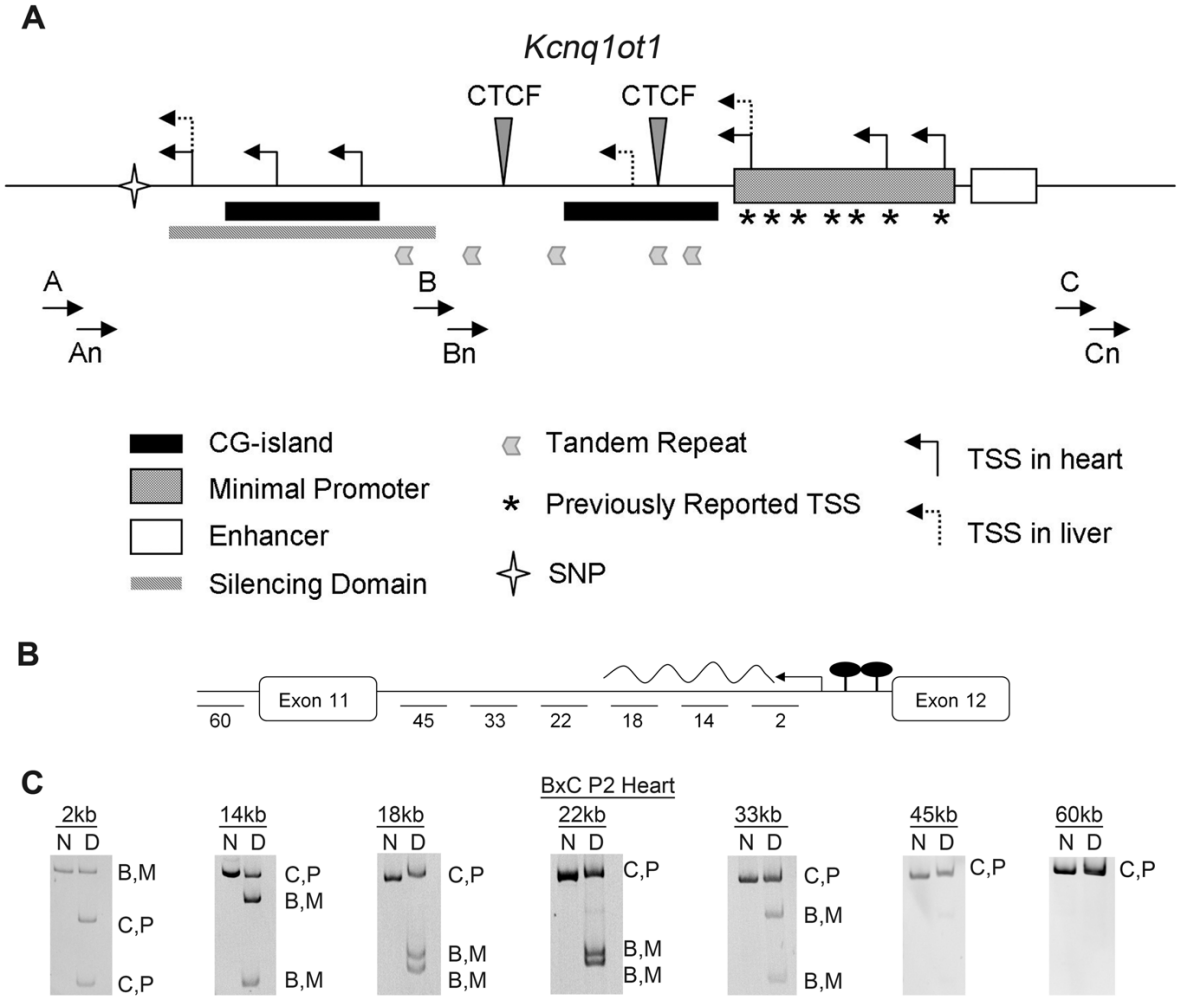
Characterization of the maternal *Kcnq1ot1* transcript. A) Schematic showing the regulatory sequences at the *Kcnq1ot1* locus. Minimal promoter, enhancer and previously reported transcriptional start sites (*, TSS) are from Fitzpatrick *et al.* ([21]. The silencing domain depicted was characterized by Mohammad *et al.* ([23]. Primers used for 5′ RACE experiments in this report are designated A, B, C and nested primers, An, Bn and Cn. Transcriptional start sites (TSS) in heart and liver are depicted as bent arrows. A star indicates the relative location of a single nucleotide polymorphism (SNP) used to discriminate between parental transcripts. B) Schematic of the scan to determine the length of the *Kcnq1ot1* maternal transcript, with primers amplifying fragments located at the indicated distances relative to the *Kcnq1ot1* transcriptional start site. C) RT-PCRs on RNAs extracted from hearts of F1 hybrid progeny from a BxC cross, followed by allele-specific digestion, showing the maternal transcript absent after 33 kb. M, maternal; P, paternal; ND, non-digested; D, digested; B, C57BL/6J; C, B6(CAST7).

To determine if the maternal *Kcnq1ot1* transcript is initiated at an alternative promoter, thus bypassing methylation on the maternal KvDMR, 5′RACE was performed in neonatal heart (Figure 2A). We find that in the neonatal heart, transcripts are initiated at previously reported transcriptional start sites (TSS) [21] and at two additional downstream sites (Figure 2A). Sequence analysis of an expressed single nucleotide polymorphism shows that although paternal transcripts are produced from both previously TSS and the newly identified TSS, the maternal transcripts are exclusively initiated at the cardiac-specific sites downstream of the CG-islands, thus bypassing the methylation mark.

To see if the maternal *Kcnq1ot1* gene produces a full-length RNA, we scanned the region with allele-specific RT-PCR. As shown in Figure 2, the re-activated maternal transcript is truncated, producing an RNA of less than 44 kb long (Figure 2B and 2C, Figure S1B).

### *Kcnq1* expression levels and loss of imprinting are subject to genetic background effects

To determine the differences in genetic background effects on expression levels and the timing of the mono- to biallelic switch, we assayed relative mRNA abundance in B×C and C×B hearts during development (Figure S5). Early maternal expression of *Kcnq1* is higher from the CAST/EiJ than from the C57BL/6J and continues to have a relatively higher expression level throughout development (Figure S5B). Also, the C×B mono- to biallelic transition occurs two days later than in the reciprocal cross (Figure S5A), with the C57BL/6J paternal activation beginning at 16.5 dpc. In addition, the *Kcnq1* expression level attained by the paternal copy is 75% of the maternal allele in the C×B cross, whereas maternal and paternal expression is equalized in the reciprocal cross (Figure S5C). These results show that there is a strong strain-specific genetic component on chromosome 7 that determines levels of *Kcnq1* and affects the timing of paternal allele reactivation.

### *Kcnq1* is imprinted in the absence of Kcnq1ot1 in the early stage heart

Our data show that in the context of the neonatal heart, *Kcnq1ot1* does not silence *Kcnq1*. During early development, however, *Kcnq1* exhibits monoallelic expression and it was assumed that *Kcnq1ot1* was responsible for repressing *Kcnq1* in *cis* (Figure S5A). To test this assumption, we examined a mutant mouse model in which the *Kcnq1ot1* transcript is severely truncated due to the inclusion of a transcriptional termination site 1.5 kb downstream of the transcriptional start site [8]. In this mouse, designated as *K-term*, transmission of the mutation from males results in loss of paternal silencing of genes in the *Kcnq1* domain in the placenta and late embryonic stages. We examined whether the absence of *Kcnq1ot1* leads to biallelic *Kcnq1* cardiac expression from embryonic day 10.5 to postnatal day 2.

**Figure 3:**
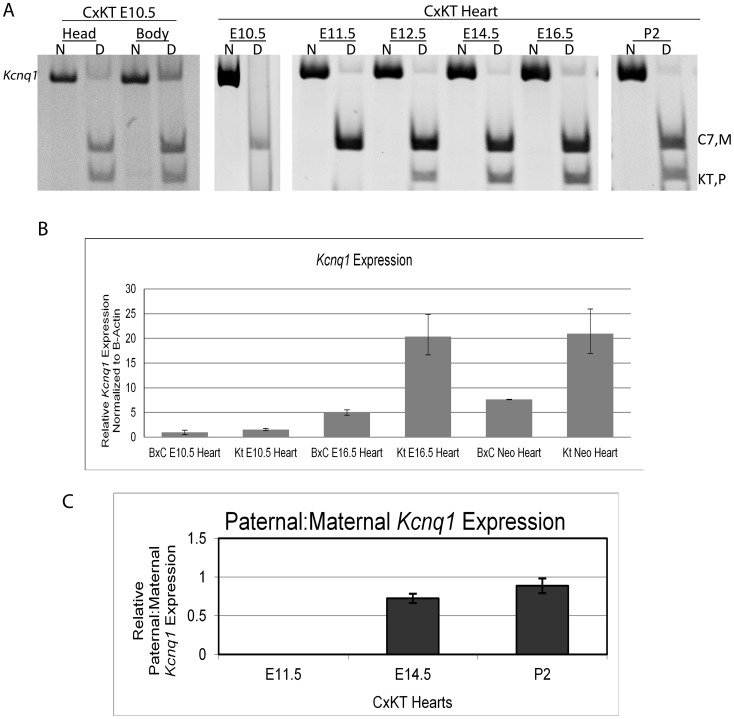
*Kcnq1* expression in the heart during development when the truncated *Kcnq1ot1* (*K-term* mutation) is inherited paternally. A) RT-PCR followed by allele-specific digests in E10.5 heads and bodies and throughout the development of the heart in F1 hybrid progeny of B6(CAST7)×*K-term* mice. M, maternal; P, paternal; N, non-digested; D, digested; B, C57BL/6J; KT, *K-term*. B) qRT-PCR analysis of *Kcnq1* expression in wild-type and *K-term* mice. Transcripts were normalized to *β-actin*. A significant difference in expression was seen when comparing wild-type and *K-term* hearts at E16.5 and P2 Hearts. These differences had a p-value less than 0.05. C) Parental origin of *Kcnq1* expression throughout cardiac development. The RT-PCR and allele specific bands were quantified and the ratio of paternal to maternal transcript was determined.

Surprisingly, *Kcnq1* did not lose imprinted expression and maintained the same profile in the *K-term* as in wild-type mice, i.e., expression was exclusively from the maternal allele in E10.5 hearts. Thereafter, expression became biallelic, as in wild-type hearts, although at a slightly earlier E12.5 dpc (Figure 3A). These results indicate that paternal-specific repression of *Kcnq1* in early embryogenesis is independent of *Kcnq1ot1* transcription and suggest that there are other mechanisms responsible for differential expression of the *Kcnq1* gene.

To determine if the maintenance of imprinting at *Kcnq1* was specific to cardiac tissue, we tested *Kcnq1* allelic expression in heads and bodies of the matched mutant embryos. In these, biallelic expression of *Kcnq1* was seen in the absence of full-length *Kcnq1ot1* (Figure 3A, left), indicating that maintenance of imprinted expression in *K-term* mutants may be restricted to the heart. These results also show that while the ncRNA is not responsible for imprinted expression of *Kcnq1* in the heart, it does have a role in silencing *Kcnq1* in tissues other than heart.

While imprinted expression of *Kcnq1* in the early heart is independent of *Kcnq1ot1*, paternal silencing of both *Cdkn1c* and *Slc22a18* does depend on *Kcnq1ot1* transcription: upon paternal transmission of the *K-term* mutation, these genes lose their imprinting pattern and are biallelically expressed (Figure S3).

### *Kcnq1* monoallelic expression is not due to methylation of its promoter

To determine if early silencing of the paternal *Kcnq1* allele is due to methylation of the promoter, bisulfite mutagenesis sequencing of the promoter was performed on E10.5 and P2 wild type hearts. Results show that the *Kcnq1* promoter is not methylated in the early embryonic heart and thus, imprinted expression cannot be attributed to differential methylation (Figure S6). These results confirm our published 5-methylcytosine ChIP data in E7.5 whole embryos confirm that the CG-island at the *Kcnq1* promoter is not methylated at any stage during development [18], as well as previous reports from others [22], [23].

### Absence of *Kcnq1ot1* leads to *Kcnq1* overexpression in the heart

Although *Kcnq1ot1* is not responsible for *Kcnq1* imprinted expression in the heart, we investigated if it had any repressive effect on *Kcnq1* in later stages of heart development. To test this, we determined *Kcnq1* mRNA abundance by qRT-PCR was performed on E10.5, E16.5 and P2 hearts in wild-type and *K-term* mice. *Kcnq1* transcript levels were similar between wt and *K-term* hearts at E10.5. However, by E16.5, *Kcnq1* abundance was significantly increased in *K-term* compared to wild type mice, indicating that *Kcnq1ot1* transcription plays a role in regulating *Kcnq1* levels (Figure 3B, Figure S5B).

To determine if the increase in mRNA levels of *Kcnq1* in the mutant mice was due to increased transcription from one or both parental alleles, relative transcript abundance was determined in 14.5 dpc and neonatal hearts. Activation of the paternal *Kcnq1* allele was progressive and levels reached 88% of the maternal allele RNA abundance (Figure 3C, Figure S5C). Thus, the overall increase in *Kcnq1* expression follows the same dynamics as in the wild-type heart, i.e., the paternal allele becomes progressively activated and reaches levels similar to the maternal RNA. This activation coincides with tissue-specific enhancer-driven expression in the wild-type mouse.

### *Kcnq1ot1* modulates three-dimensional chromatin conformation

We have previously shown that the *Kcnq1* promoter interacts with an upstream region in a cardiac-specific fashion [18]. Moreover, this interaction is established during the transition from mono- to biallelic expression of *Kcnq1*. Because the loss of the ncRNA results in a large increase in *Kcnq1* transcript in the heart, we investigated whether the three-dimensional conformation of the locus was altered in the *K-term* mice.

**Figure 4:**
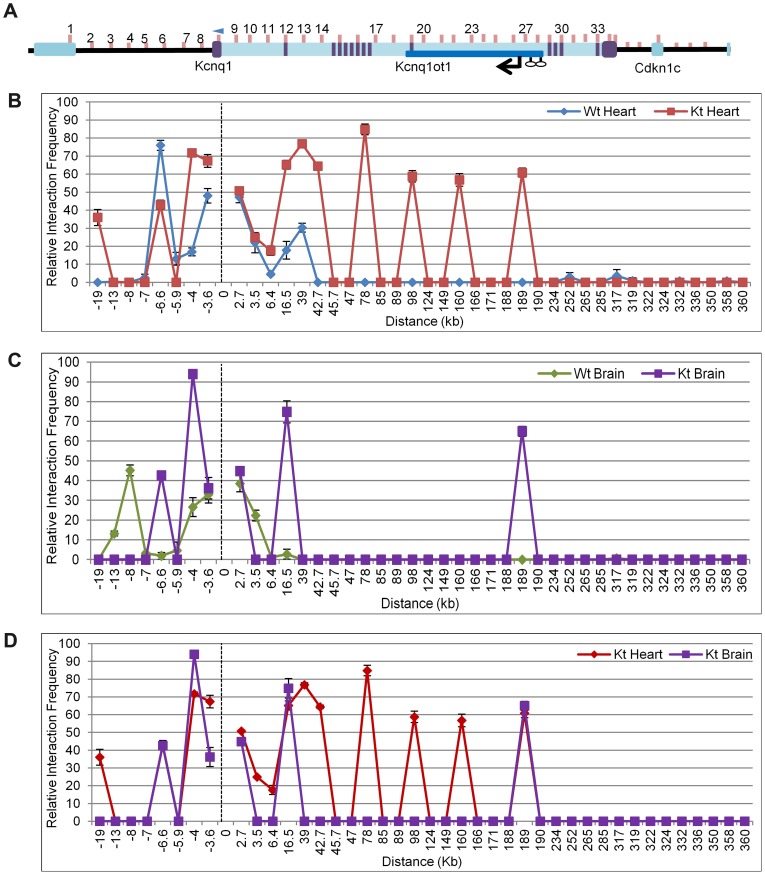
Chromosome conformation capture (3C) in the *K-term* mouse. A) Schematic of the *Kcnq1* domain. The blue arrowhead indicates the anchor primer at the *Kcnq1* promoter and vertical lines represent the regions investigated. The purple boxes represent *Kcnq1* exons, the dark blue box represents the *Kcnq1ot1* gene. Comparison of the chromatin interaction profile in wild-type and *K-term* hearts (B), wild-type and *K-term* brains (C) and *K-term* hearts and brains (D).

Chromosome Conformation Capture (3C) assays were performed on P2 hearts of *K-term* mutant mice, with the anchor primer located at the *Kcnq1* promoter (Figure 4A). In contrast to the tight interactions seen in the wild-type heart, the 3C assays showed that the *Kcnq1* promoter interacts with many additional sites throughout the region in the *K-term* mutants (Figure 4B). These results implicate the transcription of the *Kcnq1ot1* RNA in regulating the specificity of promoter interactions. Thus, although the *Kcnq1ot1* RNA does not silence *Kcnq1*, its absence is permissive for ectopic promoter contacts that increase the overall levels of *Kcnq1.*

The 3C assays are targeted to regulatory elements and there is a paucity of polymorphisms in these regions. We were able to determine allele-specificity of contacts with one primer set (primer 27) by analyzing substrate prepared from heart from progeny of crosses between B6(CAST7) and *K-term* mice (Figure S7). PCR followed by allele-specific restriction digest showed ectopic contact between the *Kcnq1* promoter and the *Kcnq1ot1* promoter region only on the paternal chromosome, i.e., the one carrying the *K-term* mutation.

Analysis of brains from *K-term* mice showed a distinct pattern, with two associations that were not present in wild-type brains. These results suggest that the biological consequences of aberrant *Kcnq1* promoter contacts are tissue-specific and reinforce that the *Kcnq1* gene has multiple levels of regulation in the heart (Figure 4C).

**Figure 5:**
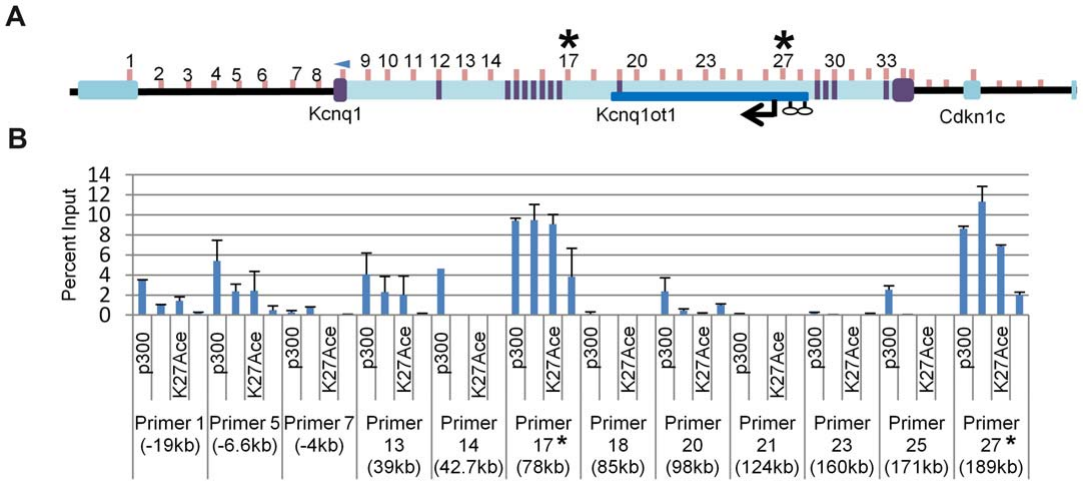
Chromatin immunoprecipitation (ChIP) of selected regions of the *Kcnq1* gene. A) Schematic of the *Kcnq1* domain. The blue arrowhead indicates the anchor primer at the *Kcnq1* promoter and vertical lines represents the primer regions investigated. The purple boxes represent *Kcnq1* exons, the dark blue box represents the *Kcnq1ot1* gene. B) ChIP analysis in wild-type heart for p300, H3K4Me1 and H3K27Ac at the interaction peaks observed in the 3C assays. The asterisks indicate frequent interactions in the *K-term* heart as determined by 3C.

To determine if the ectopic contact sites for the *Kcnq1* promoter in *K-term* mice have the hallmarks of *cis*-acting regulatory sequences, we conducted chromatin immunoprecipitation (ChIP) assays (Figure 5A, 5B). Enhancers are typically occupied by p300 and have specific epigenetic marks, notably H3K4Me1 and H3K27Ac. In addition, some enhancers are highly conserved. Using ChIP analysis, the *Kcnq1* region was scanned for p300, H3K27Ac and H3K4me1 occupancy in wild-type hearts, focusing on the sequences that exhibited interactions in the 3C assays.

Several of the ectopic contact sites showed epigenetic hallmarks of regulatory sequences (Figure 5B). Specifically, regions amplified with primer sets 13 and 17 (+39 kb and +78 kb relative to TSS, respectively) have p300 occupancy, with H3K4Me1 and H3K27Ac modifications. Although we did not observe these features with primers sets 23 and 25, H3K4Me1 cardiac-specific peaks are reported by the ENCODE consortium (annotated on UCSC), as are the primer 13 and 17 signals. Only regions 13, 21 and 23 are highly conserved (www.dcode.org), and these only between mouse, rat, rhesus and human.

Interestingly, the +189 kb peak lies within the *Kcnq1ot1* promoter region. In fact, the *Kcnq1* promoter does not make contacts beyond a *CTCF* binding site upstream (located in the *Trpm5* gene) and two *CTCF* binding sites downstream in the *Kcnq1ot1* promoter, suggesting that these may serve as boundaries to the movement of the *Kcnq1* promoter, at least in the absence of *Kcnq1ot1*.

## Discussion

The findings presented in this report underscore the value of tissue- and stage-specific studies of imprinted domains and have yielded several surprising insights. Our data and studies of other imprinted genes confirm that loss of imprinting in specific tissues is a normal developmental event. We find that strain-specific effects influence expression levels and timing of imprinting loss. We also find two co-existing mechanisms responsible for imprinting within the *Kcnq1* domain in the embryo. Importantly, by mapping the higher order structure of a specific region, we reveal a novel mechanism by which an antisense non-coding RNA can regulate transcription.

*Kcnq1ot1* is ubiquitously expressed and it had been assumed that it was imprinted in every tissue. In this study, we find that *Kcnq1ot1* is biallelically expressed in the heart, with the transition from mono- to biallelic occurring in parallel with the *Kcnq1* switch. This loss of imprinting is not seen in other tissues at that stage, indicating that there are mechanisms that shift the control of the region to meet tissue-specific needs. Perhaps the strong cardiac enhancers acting on *Kcnq1* also exert influence on the *Kcnq1ot1* promoter, activating the previously silent maternal allele.

The maternal methylation mark encompassing the *Kcnq1ot1* promoter is not lost during the activation of transcription. This is in line with abundant data showing that gametic methylation is very stable [24], and suggests that the repressive effect of DNA methylation can be overcome. In fact, our data show that maternal transcription of *Kcnq1ot1* ncRNA is initiated at an alternative promoter region downstream of the methylation mark. Several examples of alternative promoter usage bypassing DNA methylation have been reported [25], and we propose that this mechanism may be commonly deployed to fine-tune expression of imprinted genes for tissue-specific needs.

Interestingly, the *Kcnq1ot1* molecule produced from the maternal chromosome is half the length of the paternal transcript. This could be because expression of maternal *Kcnq1* from early stages blocks or competes with the *Kcnq1ot1* in some way and impedes completion of the transcript. Another possibility is that an alternative transcriptional termination signal is used. These two mechanisms are not mutually exclusive. Surprisingly, the reactivated ncRNA does not repress *Cdkn1c*, even though it accumulates locally in a manner similar to the paternal *Kcnq1ot1*. It remains to be determined if this is because of the diminished length of the RNA or if cardiac cells do not provide repressive co-factors that are necessary for the spread of silencing.

Our data reveal a genetic background effect on the level of expression of *Kcnq1*, with the CAST/EiJ allele exhibiting higher abundance than the C57BL/6J allele at all stages in the developing heart. In addition to being expressed less abundantly, a C57BL/6J paternal allele is reactivated at a later time point than a CAST/EiJ one. Whether these two features are related needs to be determined, but our own analyses and publicly available genomic data lead us to hypothesize that strain-specific *cis*-regulatory polymorphisms may explain both phenomena. For example, sequence differences in transcription factor binding or affinity for enhancers could determine higher levels of *Kcnq1* expression from the CAST/EiJ allele, and greater accessibility to reactivation when inherited paternally.

In exploring the cardiac regulation of the *Kcnq1* gene, our findings delineate two phases, independently of strain-specific differences: one, in early development, when *Kcnq1* exhibits imprinted expression independently of *Kcnq1ot1*; and two, in the neonate, when *Kcnq1* is biallelic and its expression levels are modulated by *Kcnq1ot1*.

**Figure 6:**
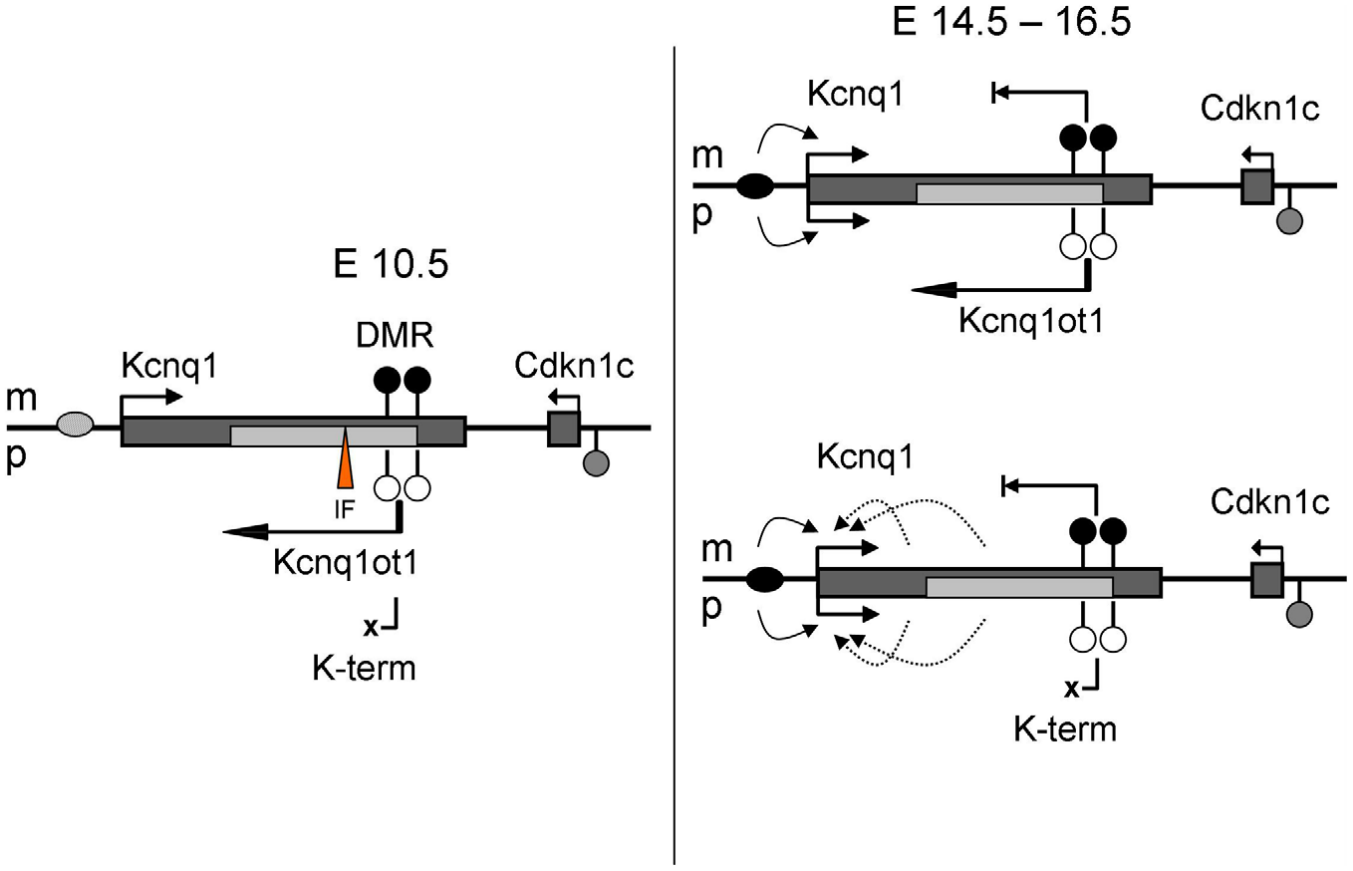
Model for regulation of *Kcnq1* in the embryonic heart. WT, wild-type; *K-term*, mutant mouse, in which transcription of *Kcnq1ot1* is terminated prematurely; IF, methylation sensitive inhibitory factor. Maternal (m) events are shown above and paternal (p) events below the chromosome; filled circles, methylated DNA, empty circles, unmethylated DNA. Curved arrows represent interactions, bent arrows depict transcription. Ovals represent enhancers, which are inactive (light gray) at 10.5 dpc and active (black) at 14.5–16.5 dpc.

Our analysis of the *K-term* mutant mouse shows that *Kcnq1* expression is imprinted in early cardiac development even in the absence of the ncRNA. This result excludes a role for *Kcnq1ot1* in establishing or maintaining repression. Thus, there is an independent mechanism that silences the paternal *Kcnq1* allele during early development. This mechanism is cardiac-specific, because absence of *Kcnq1ot1* does release *Kcnq1* from repression in tissues other than the heart. Although a secondary methylation mark at *Cdkn1c* is reportedly dependent on expression of *Kcnq1ot1*, the *Kcnq1* CG-rich promoter is never methylated. Thus, monoallelic expression of *Kcnq1* is likely due to chromatin conformation or trans-acting factors. Perhaps paternal expression of *Kcnq1ot1* opens the chromatin and makes a tissue-specific silencer available to factors that repress *Kcnq1*. These factors would be present in early development and would disappear upon full maturation of the heart (Figure 6). Another possibility is that an inhibitory factor (IF) for *Kcnq1* has a cognate bind site within a differentially methylated region. If the factor is methylation-sensitive, it would only be able to bind the unmethylated paternal allele, thus rendering the paternal *Kcnq1* allele inactive. In fact, a silencing domain (SD) has been delimited downstream of the *Kcnq1ot1* promoter, overlapping one of the differentially methylated CG-islands (Figure 2A). Interestingly, our bisulfite sequencing results for the paternal KvDMR show a trend towards acquisition and spreading of methylation that could be explained by loss of the inhibitory factor binding at the SD (Figure S3).

Early paternal repression of *Kcnq1* may also be due to the presence of *CTCF* on the paternal KvDMR [21]. *CTCF* binding is methylation-sensitive. *CTCF* could negatively impact expression of *Kcnq1* in early embryonic stages by repressing it directly or blocking it from access to enhancers required at that stage (Figure 6, inhibitory factor (IF) would be *CTCF*). Enhancer activity has been shown for a sequence immediately upstream of the *Kcnq1ot1* promoter, a position that would be blocked by *CTCF* [21].

Comparison of *Kcnq1* abundance and domain conformation between wild-type and mutant mice reveals what may be the key role for *Kcnq1ot1* in later cardiac development. Our results show that absence of the ncRNA leads to much increased *Kcnq1* levels, accompanied by a wider range of promoter contacts with sequences throughout the region. Although several of the regions contacted exhibit the marks of regulatory sequences, the *Kcnq1* promoter is only associated with them if *Kcnq1ot1* is prematurely terminated. The ectopic interactions reflect a greater flexibility of the chromatin fiber in the absence of *Kcnq1ot1* transcription and may contribute to the increased *Kcnq1* levels. It is imperative to further elucidate the molecular basis of the tissue-specificity of this effect. Subnuclear localization of the *Kcnq1* domain in the presence or absence of the *Kcnq1ot1* RNA will be an interesting avenue to pursue, to determine whether the ncRNA can act to tether the region to specific nuclear compartments [1], [26], . We also need to explore whether these mechanisms are more widely used by other ncRNA, especially those that are antisense to coding genes.

Tissue-specificity of *Kcnq1ot1* repression has been previously reported, specifically for the neighboring *Cdkn1c* gene [14]. In fact, *Cdkn1c* imprinting is maintained in the kidney, liver and lung even with a truncated *Kcnq1ot1*. Several studies have also shown lineage-specific imprinting regulation in the placenta [11], [29]. Indeed, these observations collectively underscore the need to identify *cis*- and *trans*-acting factors and the dynamics of their interactions to test hypotheses relative to the tissue-specific effects of *Kcnq1ot1*.

Overall, our results are in accordance with the idea that the *Kcnq1ot1* ncRNA itself is not responsible for directly silencing neighboring genes [13]. We posit that distinct *cis*-acting elements are made available for regulatory contacts depending on whether the act of transcription occurs or not, as previously suggested [30]. These associations have an additional layer of regulation that imposes the tissue-specificity of the patterns.

In conclusion, our results paint picture of the regulation at the *Kcnq1* domain that is much more complex than previously assumed. Imprinting centers, which regulate extended domains of genes, are limited and counteracted by transcriptional mechanisms that may have evolved in response to the emergence of imprinting. Our studies on the *Kcnq1* domain underscore that the expression pattern of a domain is ultimately determined by cooperative and competitive dialogues between enhancers, silencers and insulators [31], [32] and that understanding complex genetic loci will require identifying all the regulatory sequences and determining their linear order, tissue-specificity and three-dimensional structure.

## Materials and Methods

### Ethics statement

This study was carried out on mice in strict accordance with the recommendations in the Guide for the Care and Use of Laboratory Animals of the National Institutes of Health. The protocol was approved by the Temple University Animal Care and Use Committee (Protocol 3294).

### Mouse strains and crosses

All wild-type tissues used in the experiments were either C57BL/6J or F1 progeny of reciprocal crosses between C57BL/6J and B6(CAST7) (a consomic strain harboring a chromosome 7 derived from *Mus musculus castaneus* on a C57BL/6J background) (BxC or CxB for short). For the *K-term* mouse samples, mutant males were crossed with B6(CAST7) females and tissues of F1 progeny were obtained (Cx*K-term*). To show the transition in allelic expression for *Kcnq1* and *Kcnq1ot1*, 2–3 embryos were analyzed per litter and 3–5 litters were analyzed at each stage of development.

### RNA purification

Tissues for total RNA extraction were collected at appropriate days of gestation from F1 progeny. Polymorphisms between strains were used to distinguish between parental alleles. RNA was extracted using either TRIzol Reagent (Invitrogen #15596-018) or the Roche High Pure RNA Isolation Kit (#11828665001), following the manufacturer's protocol. All RNA samples were subjected to DNAse treatment using Turbo DNA-free (Ambion #AM1907). Three to five biological samples were collected for each tissue.

Following the manufacturer's instructions, complementary DNA synthesis was performed on total RNA using SuperScript II Reverse Transcriptase (Invitrogen #18064-014). A Reverse Transcriptase negative control was used to ensure there was no DNA contamination.

### Real-time qRT–PCR

Transcript levels of *Kcnq1*, *Kcnq1ot1* and *β-actin* were analyzed on the ABI Step One Plus system. Reactions were conducted using the SYBR Green PCR Master Mix (ABI #4309155) in a final reaction volume of 20 µl. The PCR was performed under the following conditions: an initial denaturing step for 10 minutes at 95°C, an amplification step for 45 cycles of 95°C for 20 seconds, 55°C degrees for 30 seconds and 72°C for 30 seconds, and a final elongation step at 72°C for 2 minutes. The *Kcnq1* transcript was detected using the following primers: 5′-CAAAGACCGTGGCAGTAAC-3′ and 5′-CCTTCATTGCTGGCTACAAC-3′. *Kcnq1ot1* was detected using 5′-GGTCTGAGGTAGGGATCAGG-3′ and 5′-GGCACACGGTATGAGAAAAGATTG-3′. The *Kcnq1* and *Kcnq1ot1* levels were normalized to *β-actin*, using the primers 5′-TGTTACCAACTGGGACGACA-3′ and 5′-CCATCACAATGCCTGTGGTA-3′. Each qRT-PCR reaction was performed in triplicate with three biological replicates together with a negative control. The *ΔΔCT* values of the *Kcnq1* transcript were normalized to the *β-actin* transcript. The standard deviation for each ratio was determined and the error bars represent one standard deviation from the average ratio. A t-test and p-value was obtained for all comparisons.

### Allele-specific RT–PCR and quantification

The *Kcnq1*, *Kcnq1ot1*, *Cdkn1c*, and *Slc22a18* transcripts were amplified using Ruby Taq Master Mix (Affymetrix - #71191) in a 15 µl reaction. PCR reactions were performed with experimental and control templates. Following the PCR, restriction digests were performed with *Nla*III, *Stu*I, and *Taq^α^1* (New England Biolabs) respectively. PCR and digestion products were run on 7% polyacrylamide gels and quantified using the Kodak Gel Logic 2000 imaging system. The ratio of paternal to maternal band intensities was calculated for Kcnq1. The Slc22a18 product was run on a 2% agarose gel and extracted using the E.Z.N.A Gel Extraction Kit (Omega Bio-tek, Inc.) The purified PCR product was then sent for sequencing analysis by Europhins MWG Operon. Sequences were aligned with Geneious Pro 4.6.5. The following primers were used to amplify the transcripts:

*Kcnq1* 5′-CATCGGTGCCCGTCTGAACAGG-3′ and 5′-TTGCTGGGTAGGAAGAGCTCAG-3′ with *NlaIII*

*Kcnq1ot1* 5′-GGTCTGAGGTAGGGATCAGG-3′ and 5′- GGCACACGGTATGAGAAAAGATTG-3′ with *StuI*

*Cdkn1c* 5′-CGGACGATGGAAGAACTCTGG-3′ and 5′-TACACCTTGGGACCAGCGTACTCC-3′ with *Taq^α^1*

Slc22a18 5′-GTGCTGCCTGTATTCCTGGT-3′ and 5′-CAGTCCCACAACAGCAAAGA-3′

### *Kcnq1ot1* gene scan

The following primers and associated restriction enzymes were used to scan the *Kcnq1ot1* maternal transcript in P2 hearts from reciprocal crosses of C57BL/6J and B6(CAST7) mice:

2 kb *Kcnq1ot1* 5′-GGTCTGAGGTAGGGATCAGG-3′ and 5′- GGCACACGGTATGAGAAAAGATTG-3′ with *StuI*

14 kb 5′-CTGGCCATCATTCACAGTTG-3′ and 5′- CATTCCCGTAAGAGCTGGTG-3′ with *HpyCH*4IV

18 kb 5′-CAAAAAGCAGGTCCTGAAGC-3′ and 5′-TGATGCCTTGTGATGGAAAA-3′ with *Mnl*I

22 kb 5′-AGGCACAAATGAACTGAAAGA-3′ and 5′-GCACCTCCTGTGAAGTGAGA-3′ with *BceA*I

27 kb 5′-CAGTGGCTACGGACCATCTT-3′ and 5′-TGGATACCTGTCCACATTTGC-3′ with *Sml*I

33 kb 5′-TATCCCCCTGATCACTCACC-3′ and 5′-CCTCGTTGGTGCTCACAGTA-3′ with *Nco*I

44 kb 5′- TCAACTTTTGGAGAAGATAGTGCTT-3′ and 5′- AGGACAGGCAATGACAGAGC-3′ with *Spe*I

60 kb 5′-GATCAGCATGGGTTATTGGA-3′ and 5′ –ATTAAGGGACCACAGCAAGG-3′ with HpaI

### 5′ RACE assays

5′ RACE assays to determine the *Kcnq1ot1* start site were conducted using the 5′ RACE system (Invitrogen), with RNA from three independent samples of neonatal hearts and livers. Primers for cDNA synthesis and nested PCR amplification (combined with a universal adapter primer) were as follows:

A: 5′-ACTGTATTAAAGGGTCAAAGCACAA;

An: 5′-GCAACCACTGCGGCCTCCACCCCAAGTTCCCAATC

B: 5′-ATGACGAAACAAGATAAGACCTCAC

Bn: 5′-GGACAAACACTGAGGAGGATCGCGTTGAGCAAAGC

C: 5′-CTGCTCTTCCTTACTCTAAGCACTGT

Cn: 5′-TAAGCCTTTGTTTGCCTCCTCTGGCCTAGAAGGCC

Relative positions of the primers are shown in Figure 2A. Bands of interest were recovered after gel electrophoresis, cloned (TOPO-TA Cloning Kit, Life Technologies #K4500-01) and transformed into competent E.coli cells. Plasmid DNA was prepared from bacteria (Spin Mini Prep Kit (QIAGEN #30020)and sequenced (Eurofins MWG Operon). Sequences were aligned with Geneious Pro 4.6.5 [34].

### Chromosome conformation capture (3C) assays

3C was performed as described previously [18]. Digestion efficiency at each restriction site was determined by comparing amplification with primers spanning the restriction site to amplification with primers immediately downstream on the digested template. No restriction bias was observed across the region assayed by 3C [18].

PCR efficiency of each combination of primers was assessed with a control template prepared from three BACs encompassing the *Kcnq1ot1* region analyzed, as described previously. All PCR reactions were performed with RubyTaqTM (USB) as follows: 67° 20″, 72° 20″ for 15 cycles, with a decrease in the annealing temperature of 2° every 3 cycles, followed by 25 cycles of 55° 20″, 72° 20″ and a final extension step of 72° 1′30″. The linear range for each primer pair was determined by serial dilution. PCR reactions were performed with experimental and control templates in parallel and PCR products were run on 7% polyacrylamide gels and quantified using the Kodak Gel Logic 2000 imaging system. Crosslinking frequencies were calculated from duplicate PCR analyses of three independent 3C preparations. Interaction frequencies for *Kcnq1* and *Kcnq1ot1* were normalized to a control interaction to allow comparisons between different tissues and cell types. A full list of primers can be found in Table S1.

### ChIP assays

Primary tissues were collected at neonatal stage P2, chopped into 1 mm pieces and cross-linked in 1% formaldehyde for 10 minutes. The formaldehyde was quenched with 0.125 M glycine for 5 minutes. The cells were spun down and the cell pellet was washed twice in PBS before re-suspension in lysis buffer (10 mM Tris pH 7.5, 10 mM NaCl, 3 mM MgCl2, 0.5% NP-40). Nuclei were extracted from the cells by incubating on ice for 10 minutes for brain tissue and 30 minutes for heart tissue, followed by 15 strokes in a dounce homogenizer. Nuclei were washed twice with PBS and resuspended in digestion buffer (10 mM Tris pH 7.5, 10 mM NaCl, 3 mM MgCl2, 1 mM CaCl2 and 4% NP-40). 10^7^ nuclei were sonicated and 8 ug of chromatin was used for each IP, with 10% saved as the input. Protein A beads were primed with their respective antibody in IP buffer (20 mM Tris pH 8, 2 mM EDTA, 1% Triton X-100 and 150 mM NaCl) for 2 hours at 4°C. Chromatin was pre-cleared with IgG in IP buffer at 4°C for 1 hour. Chromatin and antibody/bead complexes were incubated overnight and washed 3 times with buffer #1 (20 mM Tris pH 8, 2 mM EDTA, 0.1% SDS and 150 mM NaCl), twice with buffer #2 (20 mM Tris pH 7.5, 2 mM EDTA, 1% Triton X-100, 0.1% SDS and 500 mM NaCl), once with buffer #3 (10 mM Tris pH 8, 1 mM EDTA, 0.25 M LiCl and 1% NP-40) and twice with TE. The chromatin was eluted by incubating chromatin/antibody/bead complex at 65°C for 15 minutes in a buffer containing 25 mM Tris pH 7.5, 10 mM EDTA and 0.5% SDS. A final concentration of 0.5% SDS and 1.5 ug/ul of Proteinase K was added to all samples including the input. The samples were incubated for 1 hour at 42°C and de-crosslinked overnight at 65°C in a shaking water bath, followed by phenol∶chloroform extraction and ethanol precipitation. Pellets were re-suspended in 10 mM Tris.

The ChIP substrates were amplified using the ABI step-one plus machine and Sybr green reagents using the same conditions as the qRT-PCR assays. The data was analyzed using the percent input method. A full list of primers can be found in Table S2.

### Bisulfite mutagenesis sequencing

1 ug of DNA was mutagenized using the EZ DNA Methylation-Gold Kit (Zymo Research #5005) following the manufacturer's protocol. Amplification of the *Kcnq1ot1* CpG island was performed as previously described [33]. Primers for the *Kcnq1* promoter were:

KBS-F: 5′-GGTTGGTGTATTGTAAGTGT

KBS-R: 5′- CTAACAACAATAACTACCC

PCR products were cloned into the pCR2.1-TOPO vector using a TOPO-TA Cloning Kit (Life Technologies #K4500-01). The bacterial colonies were cultured and plasmids purified using a Spin Mini Prep Kit (QIAGEN #30020) and plasmids were sent to Eurofins MWG Operon for sequencing. Sequences were analyzed with Geneious Pro 4.6.5. Data were obtained from three independent biological samples, with at least 10 clones sequenced from each.

### Primary cardiomyocyte collection and culture

Collection of cardiomyoctes was performed as described (Sreejit, et. al., 2008) with the following modifications. The hearts were incubated in Hanks Balanced Salt Solution (HBSS) for 10 minutes on ice and the cells were cultured in DMEM supplemented with 10% FBS and 2 mM L-glutamine, penicillin (100 u/mL) and streptomycin (100 mg/mL). After the fibroblasts settled, the cardiomyocytes were collected and plated directly onto 4 well #1.5 sterile chambered glass coverslips (Thermo Fisher Scientific).

### RNA and DNA FISH

Cells were fixed and permeabilized using Life Technology's FIX AND PERM kit following the manufacturer's protocol. RNA FISH was performed as previously described (Golding, et. al., 2011). The probes for hybridization were made using Life Technology's BioPrime DNA Labeling kit with fosmids WI1-2505B3 and WI1-2753P18 (CHORI) following the manufacturer's protocol. Cy3-dCTP (GE) was used for RNA detection and FITC-dUTP (Roche) was used for DNA detection. After the washes for the RNA probe hybridization, DNA FISH was performed incubating the cells with 0.01 N HCl with 0.1 mg/ml (0.01%) pepsin for 3 minutes at room temperature. The cells were rinsed with PBS and dehydrated with a series of ethanol washes. The DNA was denatured for 30 minutes at 85°C with 70% formamide in 2× SSC. The cells were rinsed with 2× SSC and the probe was hybridized overnight. The following day, excess probe was washed with SSC, mounted and stained with Vectashield augmented with DAPI (Vector Laboratories). Cells were imaged on a Leica Sp5 Confocal Microscope with Z-stacking.

## Supporting Information

Figure S1 Allele-specific analyses of *Kcnq1ot1* in F1 hybrid mice from crosses between B6(CAST7) (C) and C57BL/6J (B) mice (CxB). A) *Kcnq1ot1* imprinting pattern as determined by RT-PCR followed by allele-specific restriction digest. The primers are located 2 kb downstream of the canonical transcriptional start site. M, maternal; P, paternal; N, non-digested; D, Digested; B, C57BL/6J; C, B6(CAST7). B) Scanning of *Kcnq1ot1* RNA for progeny from CxB crosses by RT-PCRs followed by allele-specific digestion. Primers are depicted in Figure 2. Results show absence of the maternal transcript 45 kb downstream of the transcriptional start site. M, maternal; P, paternal; N, non-digested; D, digested; B, C57BL/6J; C, B6(CAST7). C) Quantitative analysis by qRT-PCR of *Kcnq1ot1* expression throughout development. RNA levels were normalized to *β-actin*.
(TIF)

Figure S2 RNA and DNA fluorescent *in situ* hybridization (FISH) against *Kcnq1ot1*. RNA (red, top left) and DNA (green, top right) FISH in primary cardiomyocytes with probes designed against *Kcnq1ot1*. The nucleus is stained blue with DAPI (bottom left). A merge of all three (bottom right) shows two signals for RNA (maternal and paternal transcripts) and that they completely overlap with the DNA signal within the nucleus. Approximately 100 nuclei were analyzed for RNA-DNA FISH expression and 74% of the nuclei analyzed were positive for two RNA signals.
(TIF)

Figure S3 Allele-specific expression of imprinted genes in the *Kcnq1* Domain. A) BxC, CxB and CxK-term *Cdkn1c* imprinting as determined by RT-PCR followed by allele-specific restriction digest in P2 Heart, Liver and Brain. M, maternal; P, paternal; ND, non-digested; D, Digested; B, C57BL/6J; C, B6(CAST7); KT, *K-term*. B) Sequencing analysis of *Slc22a18* to determine allelic expression in P2 Heart, Liver and Brain. Asterisks indicates location of polymorphism.
(TIF)

Figure S4 *KvDMR* Methylation. A) Schematic of the CG-islands analyzed by bisulfite sequencing. B) Representative methylation results for the *KvDMR* on the maternal and paternal strands of E10.5 and P2 heart and P2 brain. Filled in circles represent methylated CpGs, open circles represent non-methylated CpGs.
(TIF)

Figure S5 Allele-specific analyses of *Kcnq1* in F1 hybrid mice from crosses between B6(CAST7) (C) and C57BL/6J (B) mice (CxB). A) *Kcnq1* imprinting pattern as determined by RT-PCR followed by allele-specific restriction digest. M, maternal; P, paternal; N, non-digested; D, Digested; B, C57BL/6J; C, B6(CAST7). While the BxC cross shows a mono- to biallelic transition at 14.5 dpc, the CxB cross shows full biallelic expression at post neonatal day. B) qRT-PCR analysis of *Kcnq1* expression in BxC and CxB hearts throughout development. Transcripts were normalized to *β-actin* and compared against BxC E10.5 heart using the ΔΔCT method. C) Parental origin of *Kcnq1* expression throughout cardiac development. The RT-PCR and allele specific bands were quantified and the ratio of paternal to maternal transcript was determined.
(TIF)

Figure S6 Methylation analysis of the *Kcnq1* promoter. A) Schematic of the *Kcnq1* promoter analyzed by bisulfite mutagenesis sequencing. B) Representative methylation results for the *Kcnq1* promoter. Filled in circles represent methylated CpGs, open circles represent non-methylated CpGs, numbers represent strands with the same pattern.
(TIF)

Figure S7 Allele-Specific 3C at +189 kb to TSS. A 3C PCR was performed using a CxKT Heart substrate with the anchor at the *Kcnq1* promoter and varying primer placed 189 kb downstream. The sample was then digested to determine allele of origin. B, BAC positive control; +L, 3C product; D, Digested 3C product; -L, 3C negative control; KT, *K-term* allele; P, Paternal.
(TIF)

Table S1 3C Primers. Primers used for the 3C scan on wild type and K-term samples. The primers are listed 5′ to 3′ across the domain and were used in conjunction with the anchor primer to determine if a 3C PCR product was present.
(DOCX)

Table S2 ChIP Primers. Left and right primers used for the ChIP ABI analysis. The primers are listed 5′ to 3′ across the domain and correlate to the 3C region scanned.
(DOCX)
