# Supplementary figures and images for: The Kcnq1ot1 Long Non-Coding RNA Affects Chromatin Conformation and Expression of Kcnq1, but Does Not Regulate Its Imprinting in the Developing Heart
Source: PLoS Genet. 2012 Sep 20;8(9):e1002956. doi: 10.1371/journal.pgen.1002956 (PMC3447949; doi:10.1371/journal.pgen.1002956)

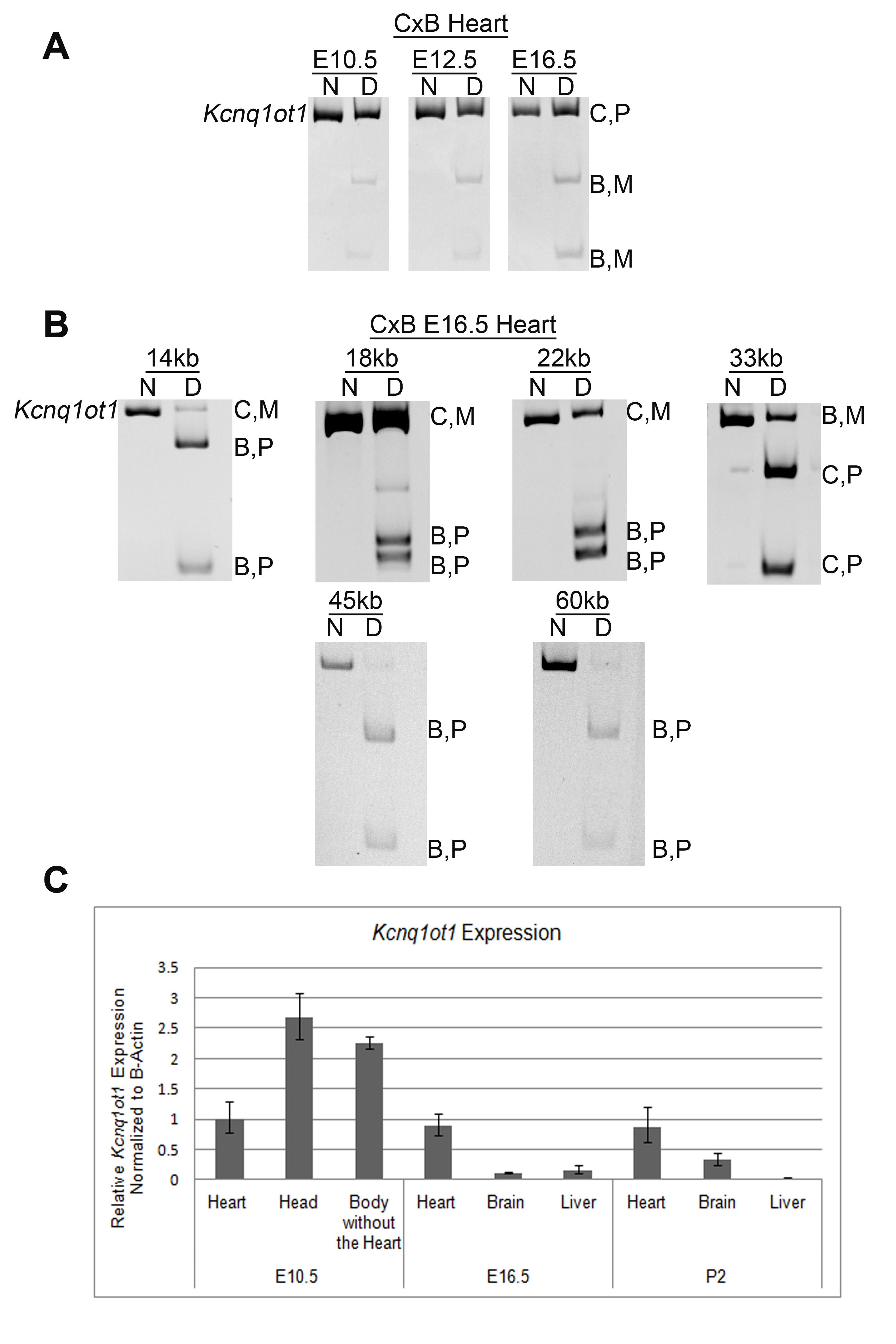

Supplement: Figure S1 — Allele-specific analyses of Kcnq1ot1 in F1 hybrid mice from crosses between B6(CAST7) (C) and C57BL/6J (B) mice (CxB). A) Kcnq1ot1 imprinting pattern as determined by RT-PCR followed by allele-specific restriction digest. The primers are located 2 kb downstream of the canonical transcriptional start site. M, maternal; P, paternal; N, non-digested; D, Digested; B, C57BL/6J; C, B6(CAST7). B) Scanning of Kcnq1ot1 RNA for progeny from CxB crosses by RT-PCRs followed by allele-specific digestion. Primers are depicted in Figure 2. Results show absence of the maternal transcript 45 kb downstream of the transcriptional start site. M, maternal; P, paternal; N, non-digested; D, digested; B, C57BL/6J; C, B6(CAST7). C) Quantitative analysis by qRT-PCR of Kcnq1ot1 expression throughout development. RNA levels were normalized to β-actin. (TIF) [file pgen.1002956.s001.tif]

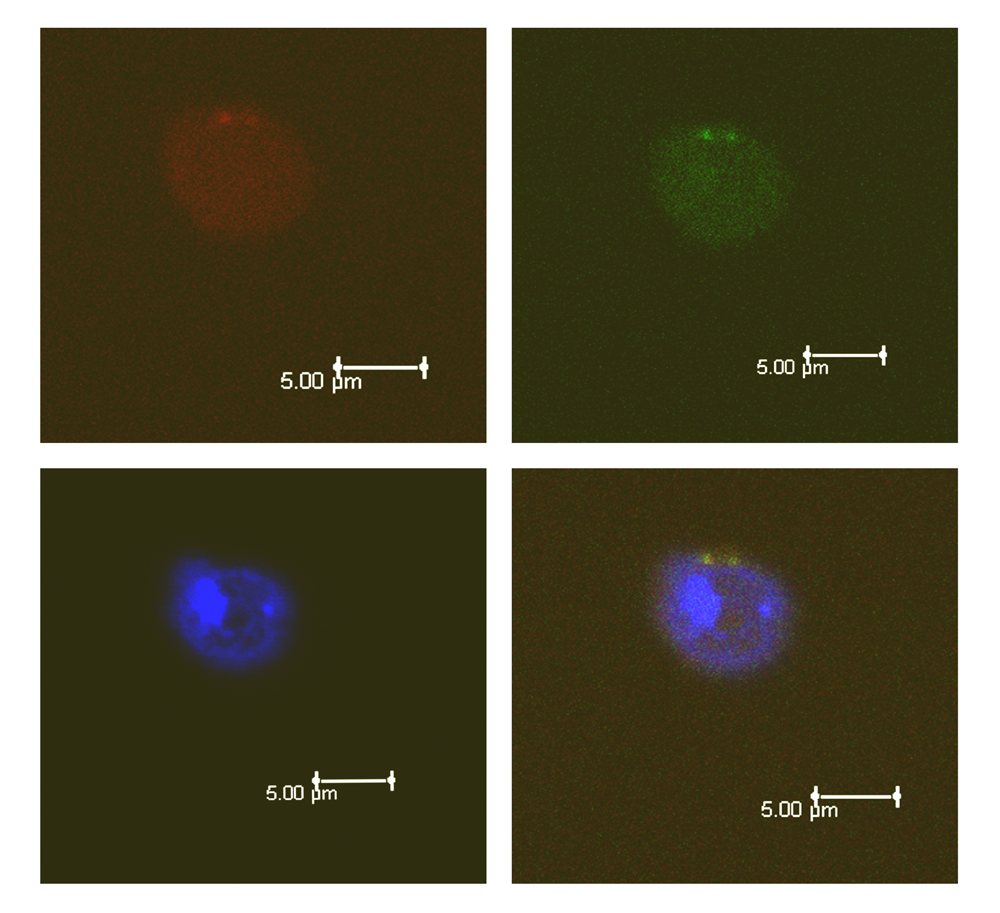

Supplement: Figure S2 — RNA and DNA fluorescent in situ hybridization (FISH) against Kcnq1ot1. RNA (red, top left) and DNA (green, top right) FISH in primary cardiomyocytes with probes designed against Kcnq1ot1. The nucleus is stained blue with DAPI (bottom left). A merge of all three (bottom right) shows two signals for RNA (maternal and paternal transcripts) and that they completely overlap with the DNA signal within the nucleus. Approximately 100 nuclei were analyzed for RNA-DNA FISH expression and 74% of the nuclei analyzed were positive for two RNA signals. (TIF) [file pgen.1002956.s002.tif]

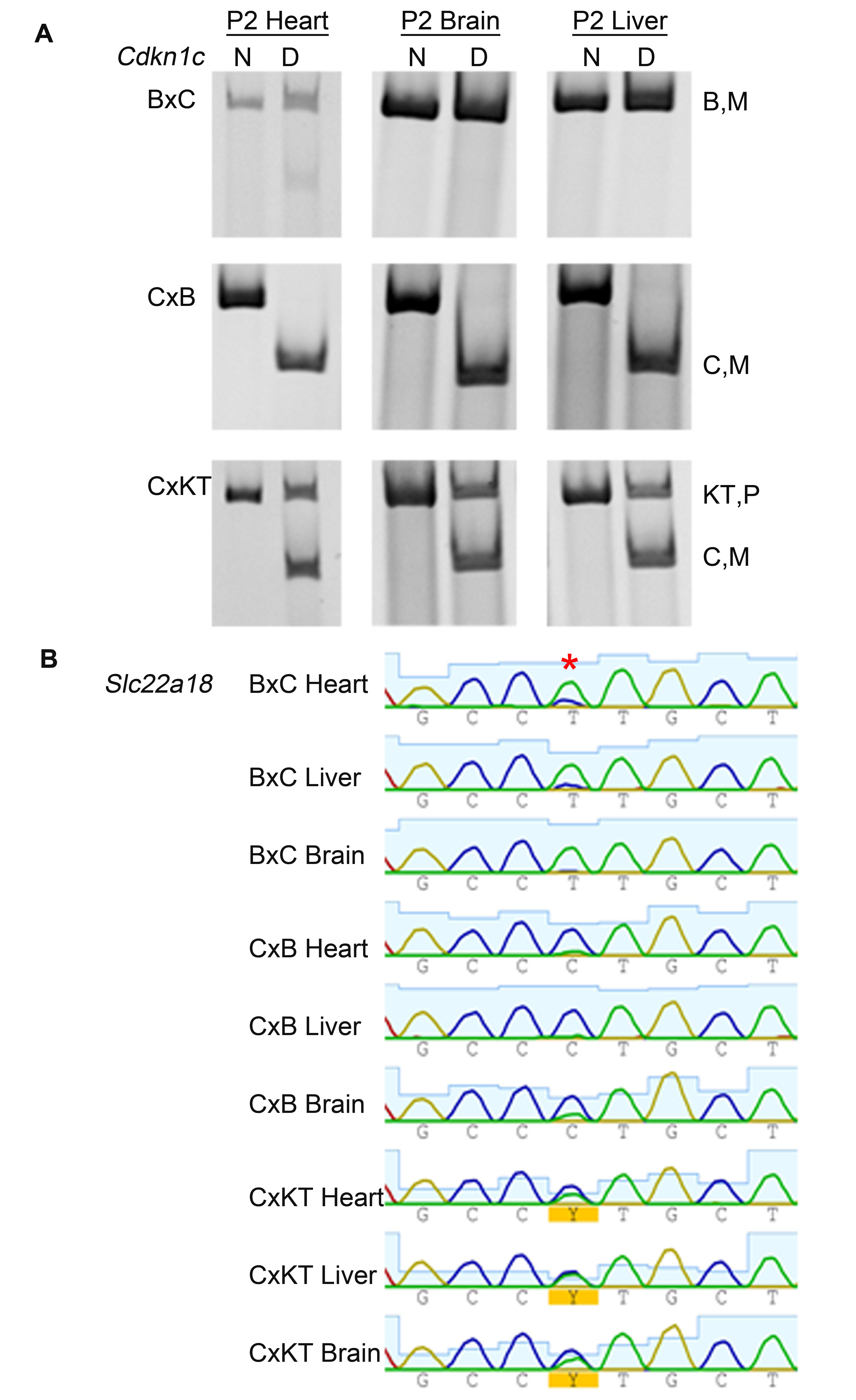

Supplement: Figure S3 — Allele-specific expression of imprinted genes in the Kcnq1 Domain. A) BxC, CxB and CxK-term Cdkn1c imprinting as determined by RT-PCR followed by allele-specific restriction digest in P2 Heart, Liver and Brain. M, maternal; P, paternal; ND, non-digested; D, Digested; B, C57BL/6J; C, B6(CAST7); KT, K-term. B) Sequencing analysis of Slc22a18 to determine allelic expression in P2 Heart, Liver and Brain. Asterisks indicates location of polymorphism. (TIF) [file pgen.1002956.s003.tif]

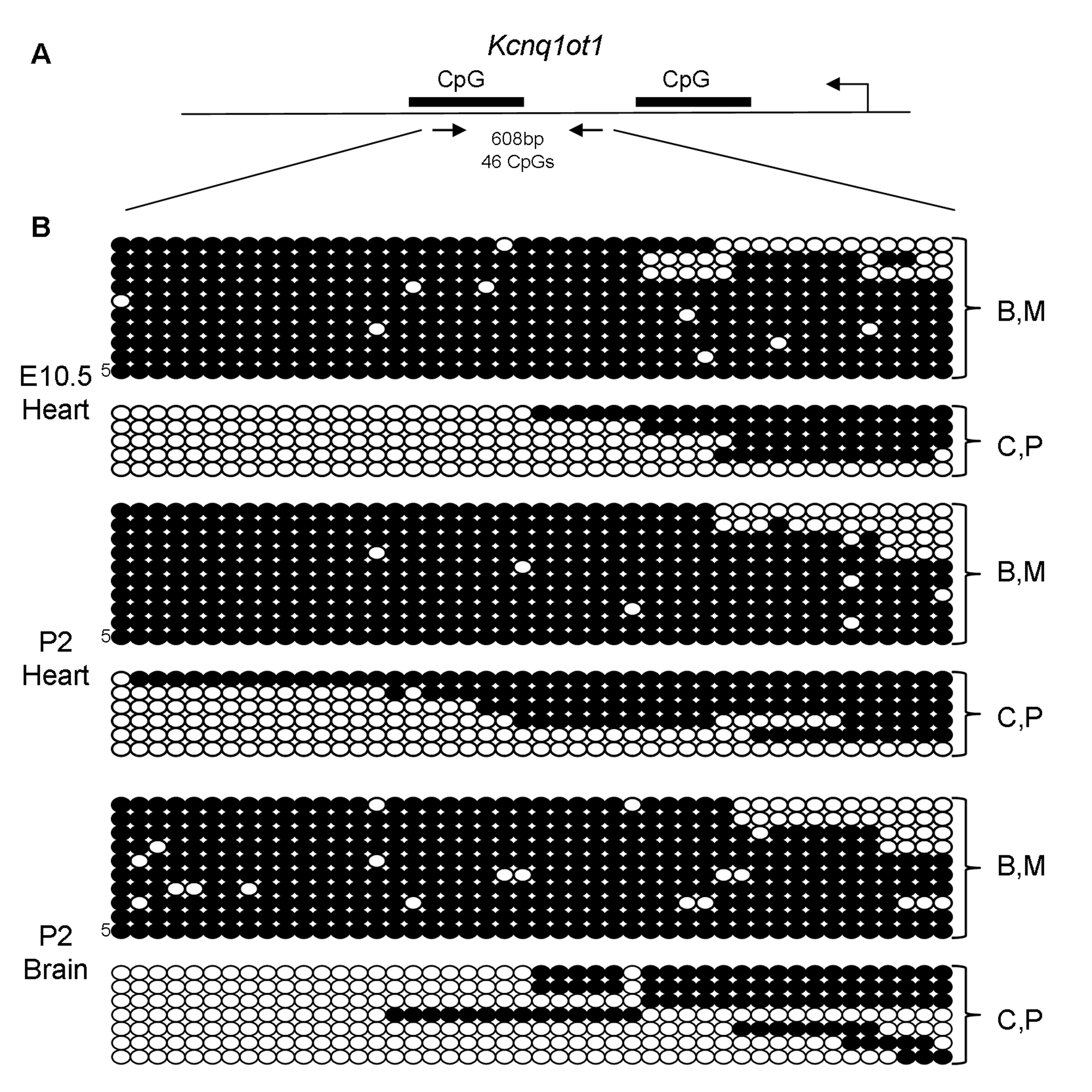

Supplement: Figure S4 — KvDMR Methylation. A) Schematic of the CG-islands analyzed by bisulfite sequencing. B) Representative methylation results for the KvDMR on the maternal and paternal strands of E10.5 and P2 heart and P2 brain. Filled in circles represent methylated CpGs, open circles represent non-methylated CpGs. (TIF) [file pgen.1002956.s004.tif]

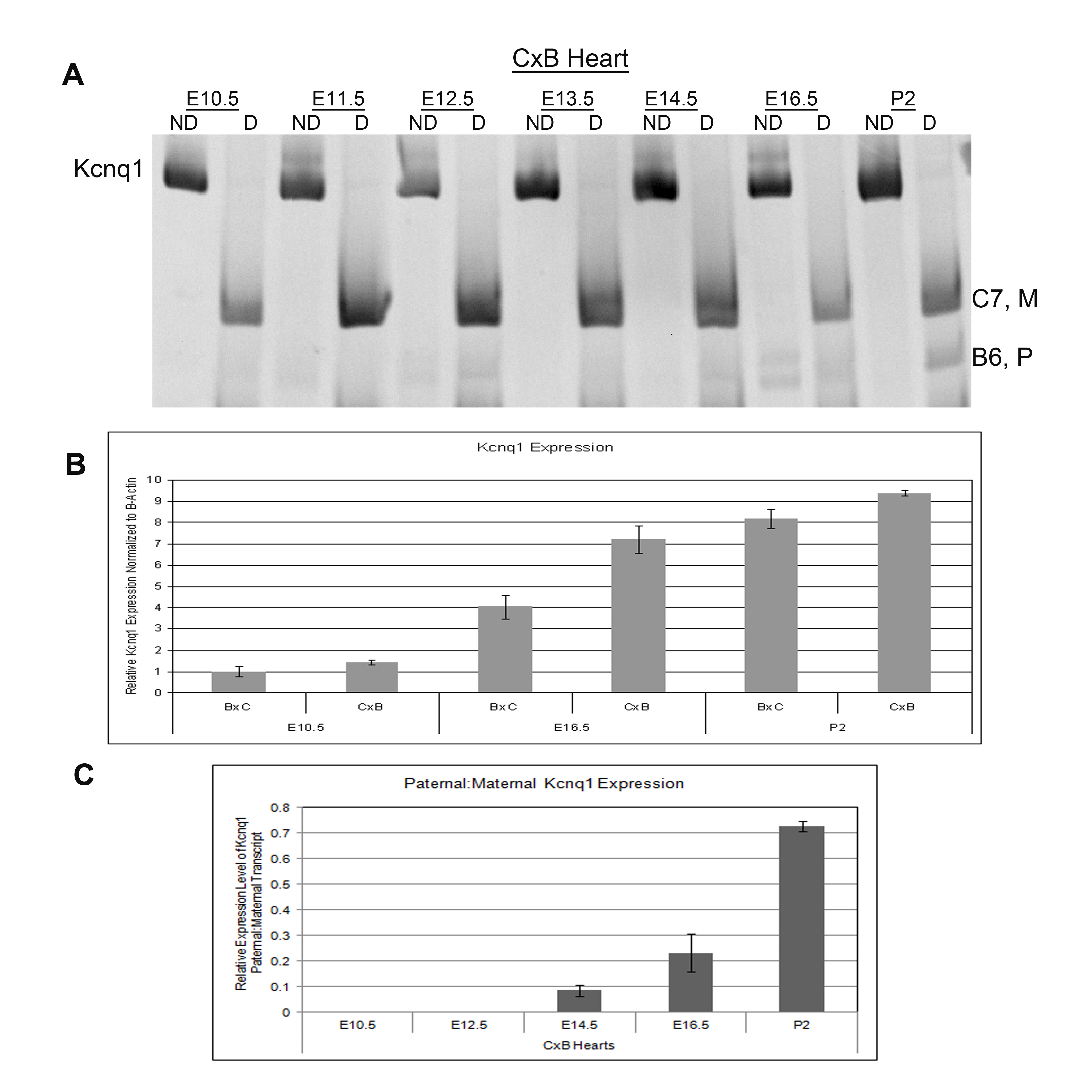

Supplement: Figure S5 — Allele-specific analyses of Kcnq1 in F1 hybrid mice from crosses between B6(CAST7) (C) and C57BL/6J (B) mice (CxB). A) Kcnq1 imprinting pattern as determined by RT-PCR followed by allele-specific restriction digest. M, maternal; P, paternal; N, non-digested; D, Digested; B, C57BL/6J; C, B6(CAST7). While the BxC cross shows a mono- to biallelic transition at 14.5 dpc, the CxB cross shows full biallelic expression at post neonatal day. B) qRT-PCR analysis of Kcnq1 expression in BxC and CxB hearts throughout development. Transcripts were normalized to β-actin and compared against BxC E10.5 heart using the ΔΔCT method. C) Parental origin of Kcnq1 expression throughout cardiac development. The RT-PCR and allele specific bands were quantified and the ratio of paternal to maternal transcript was determined. (TIF) [file pgen.1002956.s005.tif]

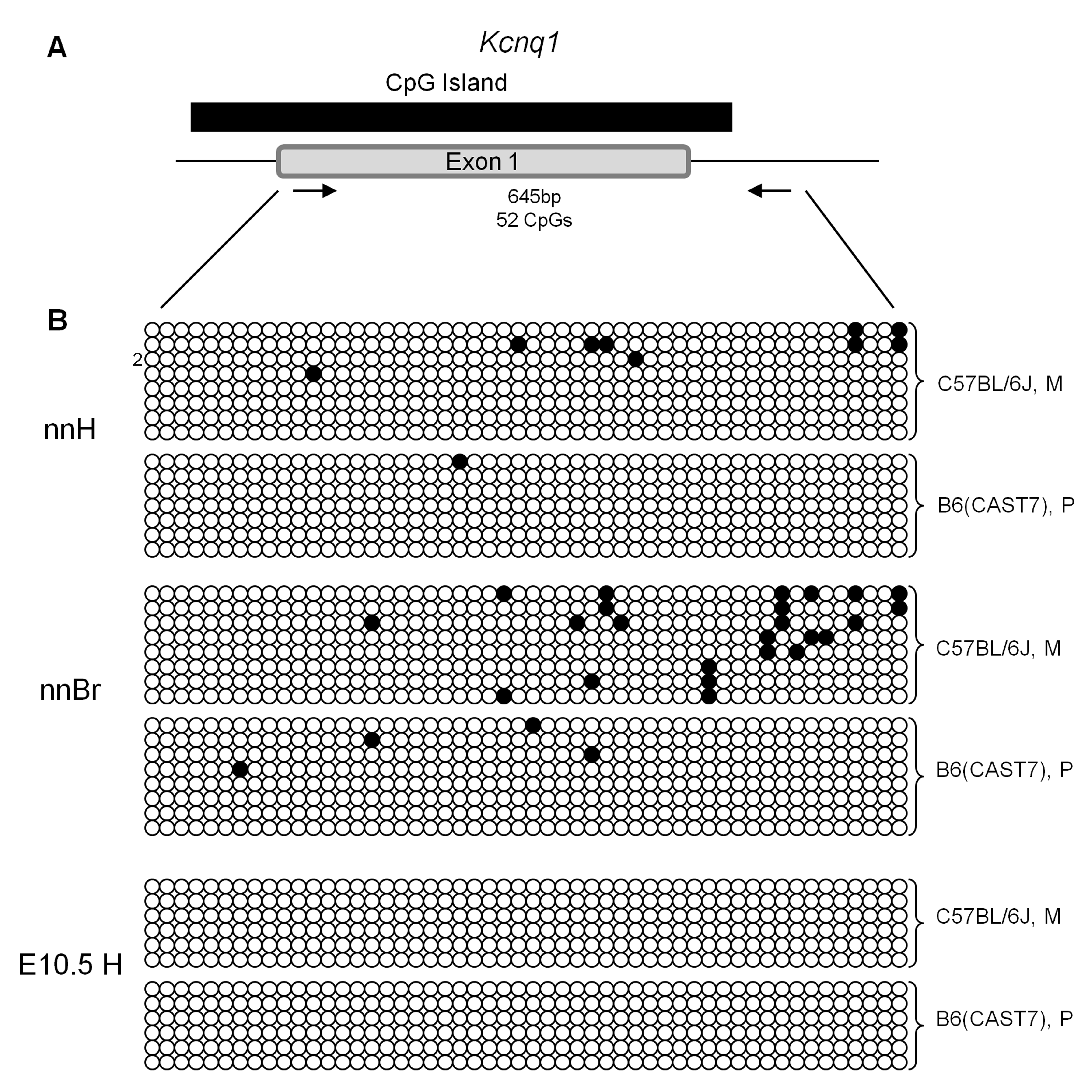

Supplement: Figure S6 — Methylation analysis of the Kcnq1 promoter. A) Schematic of the Kcnq1 promoter analyzed by bisulfite mutagenesis sequencing. B) Representative methylation results for the Kcnq1 promoter. Filled in circles represent methylated CpGs, open circles represent non-methylated CpGs, numbers represent strands with the same pattern. (TIF) [file pgen.1002956.s006.tif]

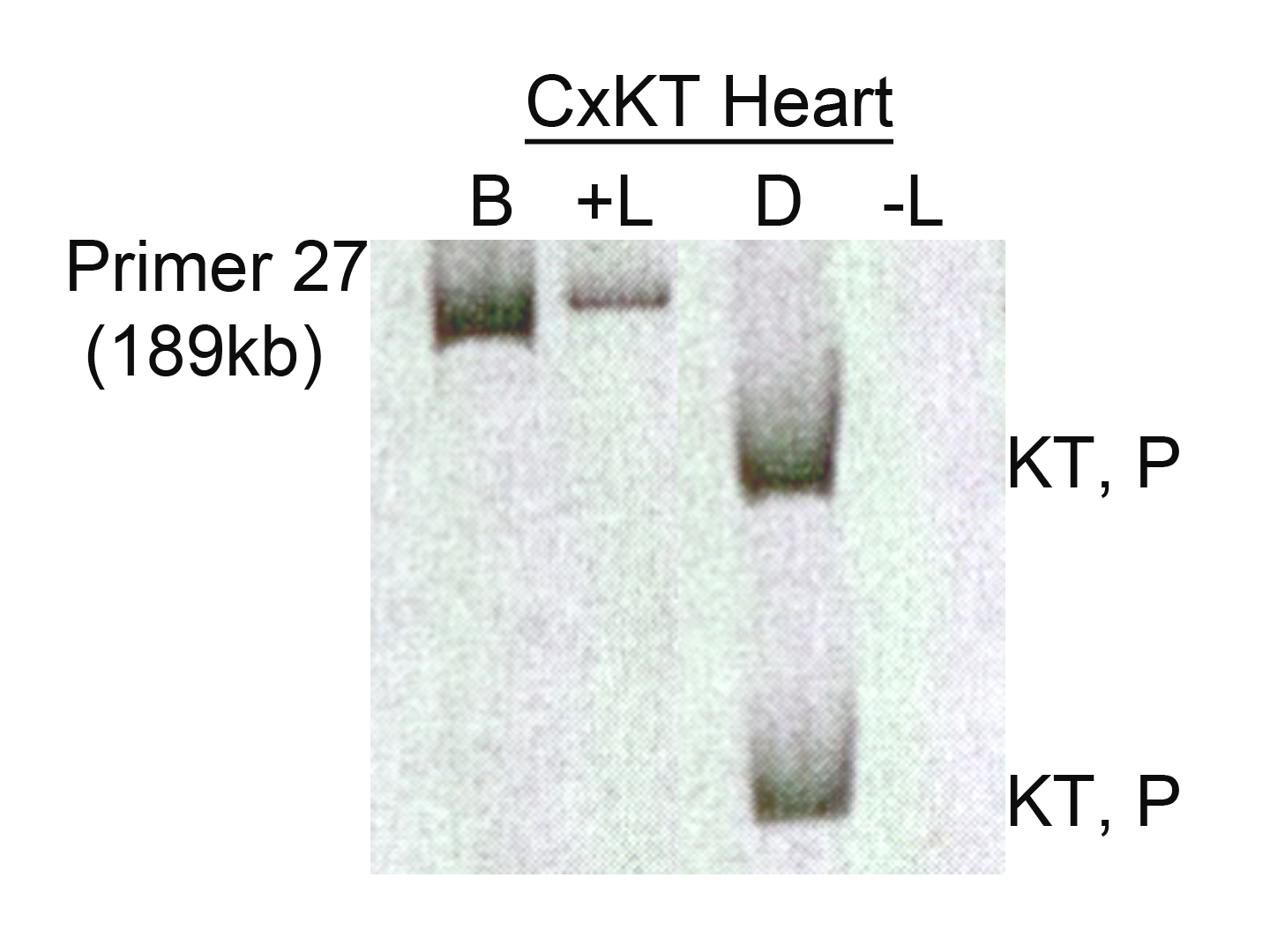

Supplement: Figure S7 — Allele-Specific 3C at +189 kb to TSS. A 3C PCR was performed using a CxKT Heart substrate with the anchor at the Kcnq1 promoter and varying primer placed 189 kb downstream. The sample was then digested to determine allele of origin. B, BAC positive control; +L, 3C product; D, Digested 3C product; -L, 3C negative control; KT, K-term allele; P, Paternal. (TIF) [file pgen.1002956.s007.tif]
