# Supplementary material for: The Kcnq1ot1 Long Non-Coding RNA Affects Chromatin Conformation and Expression of Kcnq1, but Does Not Regulate Its Imprinting in the Developing Heart
Source: PLoS Genet. 2012 Sep 20;8(9):e1002956. doi: 10.1371/journal.pgen.1002956 (PMC3447949; doi:10.1371/journal.pgen.1002956)
Supplement: Table S1 — 3C Primers. Primers used for the 3C scan on wild type and K-term samples. The primers are listed 5′ to 3′ across the domain and were used in conjunction with the anchor primer to determine if a 3C PCR product was present. (DOCX) [file pgen.1002956.s008.docx]

3C Primers

| Primer | Sequence 5' to 3' |
| --- | --- |
| Anchor | CGAAAATCTCTTCCCAGTAGTTAATC |
| 1 | AGTGAGCTTGGGTGGGTAGA |
| 2 | GGTGCCATGCTAATTCATGC |
| 3 | GAGTCTGAAGTCAACAGAGGACAG |
| 4 | TGGGGCCTTTCTGTCTATTG |
| 5 | CTCCTTACCCATAGGCCACA |
| 6 | CCACTGGTGACTATTGTCTATCCTT |
| 7 | GCTTCCTTGATAAAGCAGGAGTC |
| 8 | GTTCCCACCCTGTTCTGTTC |
| 9 | CCTGGTGGCTTCTAGATTGG |
| 10 | ATCCCTTGTGGCAAGGCTCC |
| 11 | GTCTGTAGCAGGAGTGAGTGAATG |
| 12 | TCAAGCATCTATCTATCCCT |
| 13 | GCACAGAGATATTGAAGTGATGTCC |
| 14 | TGAGGCAGACCAGAACAATG |
| 15 | ACACTGAGGGAATGGAGGAG |
| 16 | CTCCCAGAATCCCTCACAGA |
| 17 | ATCTGCGTAGCTGCCAAACT |
| 18 | AGTCACTACCATTGGCTACGG |
| 19 | GAAGAAGAAGTTCAAGCTGGATAA |
| 20 | GAAGAAGAAGTTCAAGCTGGATAA |
| 21 | TCTGGATAACGCATTGACACA |
| 22 | TCAACTTTTGGAGAAGATAGTGCTT |
| 23 | TACAAACCTACTGCATTCTCCCTAC |
| 24 | AGACAAGCTGAGCACCTGGT |
| 25 | CTGGCCTGTGAGCTAAGCAT |
| 26 | ACTGTATTAAAGGGTCAAAGCACAA |
| 27 | GAGAGAACCCAGCAGGCTAA |
| 28 | TGTTCCTCCTAGCGACAACG |
| 29 | TAATAAACCAATTACTTTGGGCAAC |
| 30 | CAGAACAAGTCCTGGATAGAATGTT |
| 31 | CGGTTGCTTCTCTTCCCTTA |
| 32 | ATGAAAGAAACAGCATTTCTGGTAA |
| 33 | CTAGACACTCTTAAGACTCTAGCCCATATC |
| 34 | TTGCTGGGTAGGAAGAGCTCAG |
| 35 | CTCTGTATGCAGGCTCAGAGGT |
| 36 | GTAAGAGAGGATAAAGCCTTATGAAACAAC |
| 37 | GAGAGGAGTCTAATGGCTAGGTGTGT |
| 38 | GACACAAGCAGCTGATCCGC |
| 39 | ATAACCCATATGATGGTAGTCTAAGTCC |
| 40 | TCTTTTAGATCTCTACAGTATTGTTCCTCA |
| 41 | GCTCCATCTTCACAGTGTCTCC |
