# Supplementary material for: The Kcnq1ot1 Long Non-Coding RNA Affects Chromatin Conformation and Expression of Kcnq1, but Does Not Regulate Its Imprinting in the Developing Heart
Source: PLoS Genet. 2012 Sep 20;8(9):e1002956. doi: 10.1371/journal.pgen.1002956 (PMC3447949; doi:10.1371/journal.pgen.1002956)
Supplement: Table S2 — ChIP Primers. Left and right primers used for the ChIP ABI analysis. The primers are listed 5′ to 3′ across the domain and correlate to the 3C region scanned. (DOCX) [file pgen.1002956.s009.docx]

ChIP Primers

| Primer | Sequence 5' to 3' |
| --- | --- |
| 1F | TTCTGGGACTTGAGGACAGC |
| 1R | AGTGAGCTTGGGTGGGTAGA |
| 5F | CTGTCCTTGGAGCAGTCACA |
| 5R | CTCCTTACCCATAGGCCACA |
| 7F | GCTTCCTTGATAAAGCAGGAGTC |
| 7R | ATACTGTTGAGCACGGAACTAAGAG |
| 13F | GCACAGAGATATTGAAGTGATGTCC |
| 13R | ATAGGGCTGAGAGCCTCAGAGAG |
| 14F | CAATCTGGGGTCAGTCCTAA |
| 14R | TGAGGCAGACCAGAACAATG |
| 17F | CCACCCTGTACATTGGCTTT |
| 17R | ATCTGCGTAGCTGCCAAACT |
| 18F | AGTCACTACCATTGGCTACGG |
| 18R | CTGGGAGTGCAAAGAAGGAT |
| 20F | GGTTCACAAATTACAAGGTTCAA |
| 20R | CCCAGTTTCCCCCACTATCT |
| 21F | TCTGGATAACGCATTGACACA |
| 21R | GGAGCAACCTTGCCTACAAT |
| 23F | TACAAACCTACTGCATTCTCCCTAC |
| 23R | TCTCAGACATCAGAGTGAATGAAAG |
| 25F | CTGGCCTGTGAGCTAAGCAT |
| 25R | CTTACGATGGCCCTAAAGCA |
| 27F | GAGAGAACCCAGCAGGCTAA |
| 27R | AGCCATCCTCAGTGTGCTTT |
